# Greater risk of incident asthma cases in adults with Allergic Rhinitis and Effect of Allergen Immunotherapy: A Retrospective Cohort Study

**DOI:** 10.1186/1465-9921-6-153

**Published:** 2005-12-28

**Authors:** Riccardo Polosa, Wael K Al-Delaimy, Cristina Russo, Giovita Piccillo, Maria Sarvà

**Affiliations:** 1 Dipartimento di Medicina Interna e Specialistica, University of Catania, Catania, Italy; 2 Department of Family and Preventive Medicine, University of California, San Diego, USA

## Abstract

Asthma and rhinitis are often co-morbid conditions. As rhinitis often precedes asthma it is possible that effective treatment of allergic rhinitis may reduce asthma progression.

The aim of our study is to investigate history of allergic rhinitis as a risk factor for asthma and the potential effect of allergen immunotherapy in attenuating the incidence of asthma.

Hospital-referred non-asthmatic adults, aged 18–40 years between 1990 and 1991, were retrospectively followed up until January and April 2000. At the end of follow up, available subjects were clinically examined for asthma diagnosis and history of allergen specific immunotherapy, second-hand smoking and the presence of pets in the household. A total of 436 non-asthmatic adults (332 subjects with allergic rhinitis and 104 with no allergic rhinitis nor history of atopy) were available for final analyses.

The highest OR (odds ratio) associated with a diagnosis of asthma at the end of follow-up was for the diagnosis of allergic rhinitis at baseline (OR, 7.8; 95%CI, 3.1–20.0 in the model containing the covariates of rhinitis diagnosis, sex, second-hand smoke exposure, presence of pets at home, family history of allergic disorders, sensitization to *Parietaria judaica*; grass pollen; house dust mites; *Olea europea*: orchard; perennial rye; and cat allergens). Female sex, sensitization to *Parietaria judaica *and the presence of pets in the home were also significantly predictive of new onset asthma in the same model. Treatment with allergen immunotherapy was significantly and inversely related to the development of new onset asthma (OR, 0.53; 95%CI, 0.32–0.86).

In the present study we found that allergic rhinitis is an important independent risk factor for asthma. Moreover, treatment with allergen immunotherapy lowers the risk of the development of new asthma cases in adults with allergic rhinitis.

## Introduction

Asthma is one of the most common chronic conditions in developed countries, with a prevalence that has been increasing globally since the 1970s [1-3]. Asthma is often associated with allergic rhinitis (AR) and the overall characteristics of the diseases and treatment options for these disorders are similar [4,5].

Several studies have suggested that AR usually precedes asthma and that rhinitis may be an important risk factor for the development of asthma. In a proportion of allergic rhinitic individuals, bronchial challenge with histamine or methacholine may reveal bronchial hyperresponsiveness (BHR) even in the absence of any asthmatic symptoms [6,7] and this may be a reflection of sub-clinical inflammatory changes in the lower airways [8-10]. Rhinitic subjects with documented BHR are known to be at risk for asthma progression [11-13]. In addition, a number of epidemiological surveys in adults suggest that allergic rhinitis may be a prelude to airway symptoms related to asthma [14-18]. However, these five studies mostly rely on postal questionnaires for the diagnosis of allergic rhinitis and asthma. Moreover, the possibility that treatment modalities (especially regular nasal corticosteroids) might have altered the natural course of the disease could not be excluded with confidence.

It is possible that effective treatment of allergic rhinitis may reduce asthma progression. The efficacy of allergen-specific immunotherapy (SIT) for allergic conditions has been highlighted in a recent World Health Organization report that advocates its use in selected patients [19]. Although the evidence of its effectiveness in asthma is still controversial, its efficacy in reducing the severity of symptoms related to allergic rhino-conjunctivitis has been established in randomized controlled studies [19,20]. It is also possible that in susceptible individuals, SIT may be effective in reducing progression to asthma rather than reversing its course once the disease is established [21-23]. However, larger studies are needed in order to confirm whether SIT is truly beneficial in decreasing the incidence of asthma in subjects with AR and to define the characteristics of patients who would benefit most from such a therapeutic approach.

The present study was carried out with a cohort of non-asthmatic adult subjects with and without AR to define its importance as a risk factor for asthma during follow-up while adjusting for known asthma risk factors. We also wanted to investigate the potential effect of SIT treatment among patients with AR in reducing asthma progression.

## Materials and methods

### Study population

**Figure 1:**
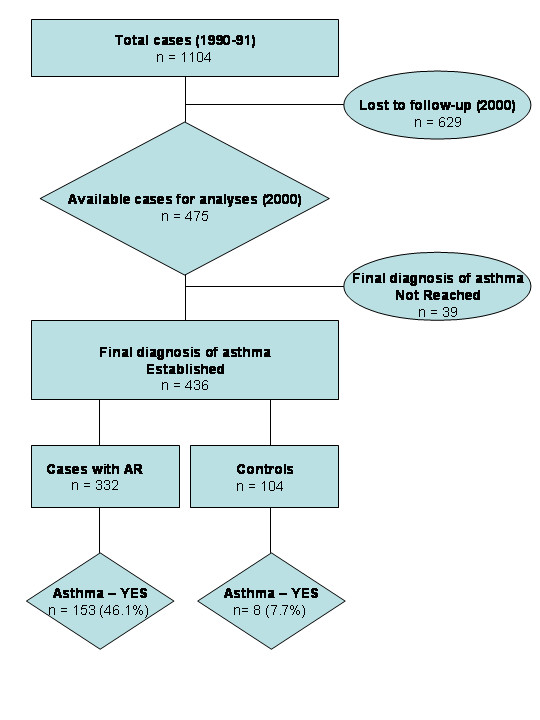
Study flow diagram. Medical records of cases who were referred in the period between January 1990 and December 1991 to the clinic for the diagnosis and treatment of allergic diseases were reviewed. To be included in the study cases had to be between the ages of 18 and 40 years and not diagnosed with asthma at the time of referral. A total of 1104 records were selected at baseline. In the period from January to April 2000, subjects were contacted for a follow up visit to evaluate the possibility of asthma diagnosis; 629 subjects were lost to follow-up leaving 475 subjects taking part in the study. A diagnosis of asthma could not be established with confidence in 39 subjects, leaving a total of 436 subjects for the final analyses. Among those 436 subjects, 332 had allergic rhinitis and 104 had no allergic rhinitis or history of atopy. At follow up, 46.1% (n = 153) of those with rhinitis at baseline developed asthma.

The Outpatient Allergy Clinic of the University of Catania, Sicily is the primary referral center for respiratory allergies in the area of Catania. We reviewed the medical records of 1104 cases, with either allergic rhinitis or with no allergic rhinitis or history of atopy, who were referred to the clinic for the diagnosis and treatment of allergic diseases (Figure 1). To be included in the initial selection the subjects had to be between the ages of 18 and 40 years and not diagnosed with asthma at the time of referral, in the period between January 1990 and December 1991. The referred cases had to be born and residing in the province of Catania – Sicily.

**Figure 2:**
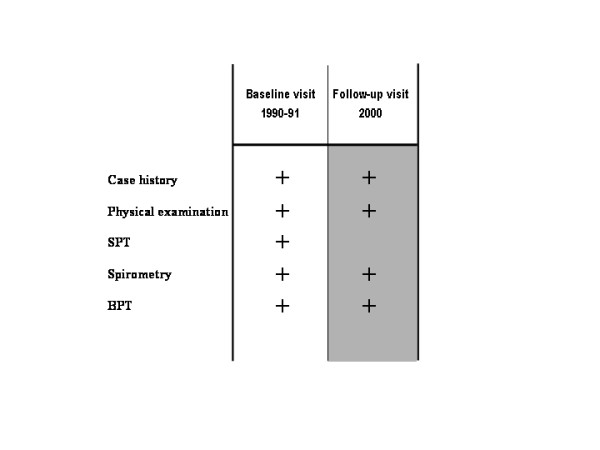
Diagnostic procedures. With the exception of skin prick testing – SPT, case history (paying particular attention to the presence of a past or present history of asthma and/or previous asthma symptoms or asthma medication intake), physical examination and simple spirometry were repeated at baseline (1990–91) and follow-up visits (2000). Bronchial provocation testing – BPT with inhaled methacholine were carried out in selected cases on both visits.

Our standardized diagnostic protocol at the time of referral consisted of case history, clinical examination, spirometry, and skin tests (Figure 2). Skin prick testing was performed on all subjects to determine sensitivity to common allergens (including *Parietaria judaic*a, *Dermatophagoides pteronyssinu*s, *Dermatophagoides farinae, Olea europea*, grass pollen, orchard, cypressus, alternaria, perennial rye, and cat allergen). We used 0.1% histamine solution as the positive quality control of the skin prick test and used the diluent media for allergens as the negative control. Skin prick tests were regarded positive if the mean wheal diameter was more than 3 mm. Referred patients who had no diagnosis of allergic rhinitis and no positive skin prick test were also included to assess the difference in asthma incidence at the end of follow up among those with and without rhinitis at the baseline. Those who were referred to the clinic, but not diagnosed with allergic rhinitis, were mainly referred because they wanted to be seen for "allergy-related" symptoms, of which most were due to drug allergy, food intolerance, chronic urticaria, post-viral rhinorrea, and infectious/viral conjunctivitis.

The diagnostic criteria used for allergic rhinitis were those defined by the Joint Task Force on Practice Parameters in Allergy, Asthma and Immunology [24] and included watery rhinorrea, nasal itch, sneezing, nasal blockage, excessive lacrimation or conjunctival redness when exposed to allergens, in combination with positive skin test reactions to suspected allergens.

Records were excluded from the study if there was a past or present history of asthma, previous asthma symptoms or asthma medication intake, and/or abnormal spirometric values at the time of referral at baseline (Figure 2). The possibility of unrecognised asthma in our study population was addressed by further reviewing their case histories and subjects were eligible for inclusion in the study only after at least two specialists in allergic diseases agreed they did not have any clinical history or symptoms suggestive of asthma. The criteria used to record a diagnosis of asthma (the main endpoint of the study) were those based on the ATS guidelines [25] and on the recommendations established by the National Heart, Lung and Blood Institute of the National Institutes of Health [26].

### Study design and procedures

The present study took the form of a retrospective cohort study of allergic rhinitic and non-allergic subjects (Figure 1). The records were selected from among the 1104 referred subjects at baseline. Subjects seeking symptomatic relief were not excluded if they only used over the counter drugs, such as topical decongestants, intranasal sodium cromoglycate, and/or oral antihistamines, when needed throughout the study follow-up. When nasal corticosteroids were prescribed, therapy had to be restricted to no more than 6 weeks/year. None of the subjects included had ever received allergen specific immunotherapy at baseline.

In the period from January to April 2000 we were able to contact 475 subjects (258 Males; 217 Females) among the study population selected at baseline (1990–1991). They were invited to the Clinic for a follow up visit in order to evaluate the possibility of asthma diagnosis [25,26] (Figure 2). All subjects reporting unclear symptoms of asthma and with normal spirometric values at the time of clinical follow up were also investigated by methacholine challenge [27] and further reviewed 9 months later for accurate classification of asthma. The remaining 629 subjects of the initial 1104 eligible subjects, (496 atopics [265 Males and 231 Females], and 133 non-atopics [56 Males and 77 Females]) were lost to follow-up because they could not be contacted as a result of extensive recoding of the local telephone lines that occurred between 1995–1996 (n = 485; 77.1%) or because they repeatedly failed to attend their follow-up visit (n = 144; 22.9%).

A diagnosis of asthma could not be established with confidence in 39 subjects (21 Males; 18 Females) at the end of follow up and were therefore excluded from the study leaving a total of 436 subjects for the final analyses. Among those 436 subjects, 332 had allergic rhinitis and 104 had no allergic rhinitis or history of atopy. On the same follow up occasion subjects were invited to complete a questionnaire on respiratory and allergic conditions (modified from the ISAAC core questions [28]), which included queries on the development of asthma symptoms, the need for drug therapy for rhinitis and/or asthma, in addition to questions on the family history for atopic disease, second-hand smoke exposure history, and pet ownership. The questionnaire also included questions on changes in the clinical rating of rhinitic symptoms if they during follow up.

The potential effect of allergen immunotherapy in decreasing the incidence of asthma was evaluated only in those subjects who had been taking allergen immunotherapy for at least 3 consecutive years during the follow up period. Allergen immunotherapy is indicated in patients with IgE-mediated disease (symptoms on exposure to relevant allergen supported by a positive SPT to that allergen) with a limited spectrum (1 or 2) of allergies and with short disease duration [19]. Patients considered for immunotherapy had documented positive skin sensitivity to at least one allergen (class ++ or more) with the duration of their rhinitis not exceeding 10 years. Allergen immunotherapy consisted of a selection of commercially available extracts of *Parietaria judaica *or *Dermatophagoides *mix (*D. pteronyssinu*s + *farinae*; house dust mites). House dust mites and *Paritaria judaica *allergen extracts conjugated with either sodium alginate (Conjuvac; DHS-Bayer) or alum hydroxide (Lofarma Depot Immunotherapy; Lofarma) or tyrosine-adsorbed glutaraldehyde-modified extract of *Parietaria judaica *pollen (Bencard Parietaria; Bencard) were generally used.

Injections were administered by trained physicians according to manufacturers' recommendations, but tailored to individual patients' clinical circumstances. In general, immunotherapy protocols involved weekly injections during an updosing phase, followed by monthly maintenance injections for a period of 3–5 years. For *Parietaria judaica *extracts a monthly maintenance dose equivalent to at least 0.48 μg of Par j1 was achieved. For house dust mites a monthly maintenance dose equivalent to at least 3.2 μg of Der 1 and 1.6 μg of Der 2 was reached.

### Statistical analyses

The data were analyzed with SAS-PC statistical package (SAS Institute, Cary, NC). The general characteristics of the study cases were described with summary statistics using frequency tables and Chi-square test. Logistic regression analysis was used to estimate unadjusted and adjusted odds ratios, significance levels, and confidence intervals for each study factor associated with asthma development. The adjusted models included all the other variables excluding IT treatment, since all those without rhinitis were not relevant to this variable. Patients on IT were either treated with Parietaria allergen or mites allergen and never included twice in the statistical calculation. The variables included in the models were: diagnosis of rhinitis at the time of the start of the study, sex, family history of allergy, pet ownership before the age of 5 years, sensitisation to a number of specific allergens, and parental smoking at home before the age of 5 years. Changes in the clinical rating of rhinitic symptoms at follow-up was separately assessed among rhinitis patients.

In order to better understand how many cases of hay fever would have to be treated with allergen IT to avert one case of asthma, the number needed to treat (NNT) was calculated.

For the analyses, 95% confidence intervals were used and p values <0.05 were considered significant. Stepwise regression analysis was carried out and only the statistically significant variables were included in the final model.

## Results

### Predictors of new onset asthma in the study population

In our study population of 436 subjects (237 Males; 199 Females) a total of 161 (36.9%) were diagnosed with asthma in 2000. Table 1 shows the data, as well as percentages for each variable at baseline in addition to the frequency, according to the diagnosis of asthma at the end of follow up. Asthmatics were more likely to have had a higher percentage of rhinitis, family history of allergic diseases, pets in the home, and sensitization to *Olea europea *and *Parietaria judaica*, all of which were statistically significant. In addition, more asthmatics were females compared to non-asthmatics. All other factors were generally higher among asthmatics compared to non-asthmatics although this did not reach statistical significance (Table 1).

**Table 1:** Frequency for each variable at baseline and distribution of covariates, at baseline and during follow up, according to diagnosis of asthma at the end of follow up.

**Covariates**	**Frequency (%)**	**Asthma**	**No-asthma**	**Chi square P value**
Presence of allergic rhinitis	332 (76.2)	*95%*	*65%*	*<0.0001*
Female sex	199 (45.1)	*57%*	*38%*	*<0.0001*
Positive family history for allergic disorders	274 (62.8)	*71%*	*58%*	*0.0085*
Presence of pets in the home	149 (34.2)	*45%*	*28%*	*0.0004*
Parental smoking at home	178 (40.8)	63%	57%	0.17
Sensitization to Parietaria judaica	232 (53.2)	*75%*	*41%*	*<0.0001*
Sensitization to house dust mite	107 (24.5)	*28%*	*23%*	*0.21*
Sensitization to Olea europea	112 (25.7)	*32%*	*22%*	*0.029*
Sensitization to grass pollen	48 (11.0)	*14%*	*10%*	*0.18*
Sensitization to orchard	100 (22.9)	*27%*	*20%*	*0.13*
Sensitization to perennial rye	99 (22.7)	26%	22%	0.34
Sensitization to cat	34 (7.8)	*9%*	*7%*	*0.37*
Sensitization to cypressus	16 (3.7)	*4%*	*3%*	*0.56*
Sensitization to alternaria	18 (4.1)	*4%*	*4%*	*0.75*

We found that 46.1% (n = 153) of those with rhinitis at baseline developed asthma at the end of follow up while only 7.7% (n = 8) of the non-allergic subjects at baseline developed asthma at the end of follow up. Severity and type of asthma have been subsequently graded according to GINA guidelines [29]. Among the 153 rhinitic subjects who were diagnosed with asthma in 2000, 46 (30%) had intermittent asthma, 91 (59.5%) had mild persistent asthma, 13 (8.5%) had moderate persistent asthma, and 3 (2.0%) had severe persistent asthma. All the non-allergic subjects with asthma at follow-up (n= 8) had mild persistent asthma.

Among subjects with allergic rhinitis, usage of nasal corticosteroids was similar at the end of follow up among the group with a diagnosis of asthma (n = 80; 52.3%) and the group who had no symptoms of asthma (n = 85; 47.5%). Presence of allergic rhinitis at the start of the study was highly predictive of development of new onset asthma after 10 years (OR, 10.3; 95%CI, 4.8–21.8) (Table 2). In the univariate analyses, the second highest OR was for family history of allergic diseases (OR 4.26 (95% CI 2.78–6.54). Variables of female gender, presence of pets, and sensitisation to *Olea europea *and *Parietaria judaica *were significantly predictive of asthma diagnosis at the end of follow up (Table 2).

**Table 2:** Odds Ratios for Factors Predicting the Development of New Onset Asthma

**Model – Univariate**		
	OR	(95% CI)

*Presence of allergic rhinitis*	*10.26*	*4.83–21788*
*Female sex*	*2.19*	*1.48–3.26*
*Positive family history for allergic disorders*	*1.74*	*1.15–2.64*
*Presence of pets in the home*	*2.08*	*1.38–3.13*
Parental smoking at home	1.32	0.88–1.97.
*Sensitization to Parietaria judaica*	*4.26*	*2.78–6.54*
Sensitization to house dust mite	1.33	0.85–2.08
*Sensitization to Olea europea*	*1.63*	*1.1–2.5*
Sensitization to grass pollen	1.52	0.83–2.77
Sensitization to orchard	1.43	0.90–2.25
Sensitization to perennial rye	1.25	0.79–1.98
Sensitization to cats	1.38	0.68–2.81
*Treatment with allergen immunotherapy*	*0.63*	*0.40–0.98*

Model – Multivariate* (significant factors only)		

*Presence of allergic rhinitis*	*7.81*	*3.05–20.04*
*Female sex*	*2.83*	*1.79–4.49*
*Presence of pets in the home*	*2.02*	*1.27–3.21*
*Sensitization to Parietaria judaica*	*2.01*	*1.16–3.47*
Treatment with allergen immunotherapy**	*0.53*	*0.32–0.86*

*Model included: Allergic rhinitis diagnosis, gender, parental smoking at home, family history of allergic diseases, presence of pets at home, sensitization to *Olea europea*, sensitization to house dust mite, sensitization to grass pollen, sensitization to orchard, sensitization to perennial rye, sensitization to cats, sensitization to *Parietaria judaica *

** Model included: immunotherapy history and all the above covariates, excluding allergic rhinitis diagnosis.

Disease duration was not predictive of new incident cases of asthma in this cohort. Those who had had rhinitis for >10 years had a non-significant OR of 1.06 (95%CI, 0.62–1.81) of developing asthma compared to those with disease duration 2-to-5 years. Likewise, those with 5-to-10 years of rhinitis had a non-significant OR of 1.30 (95%CI, 0.78–2.18) of developing asthma compared to those with a disease duration of 2-to-5 years. This observed lack of association was confirmed after adjusting for all other variables in the multivariate model (data not shown).

In the multivariate analyses, adjusting for all other covariates and using the stepwise regression analyses, the same factors as above were consistently predictive of the diagnosis of asthma, excluding *Olea europea*, which became non-significant (Table 2). None of the other allergens tested showed statistically significant associations with new onset asthma.

Among those who were diagnosed with rhinitis in 1990–1991, patients who underwent immunotherapy were more likely to report their rhinitis symptoms improving, compared to those who did not have the therapy, in a model adjusted for sex (OR 1.75; 95% CI, 1.11–2.77). Furthermore, those who reported in 2000 that their rhinitis symptoms became better were less likely to develop asthma compared to those who reported either worsening or no change in the symptoms (OR 0.33; 95% CI, 0.20–0.56). This relationship was consistent in the models that included immunotherapy and other risk factors in the model.

### Effect of allergen immunotherapy on new onset asthma

Out of the 332 allergic rhinitis subjects who were considered for analysis in the study, 202 subjects underwent allergen immunotherapy for at least 3 consecutive years (60.8%). Nineteen subjects had been treated with immunotherapy for less than 3 years and were not included in the treatment group. Usage of nasal corticosteroids was similar in both the group that used allergen immunotherapy (n = 98; 48.5%) and the group that did not (n = 67; 51.5%).

**Figure 3:**
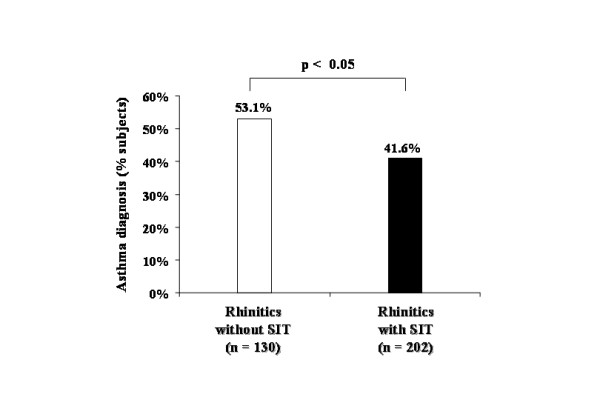
Percentage of new onset asthma by the end of the study in allergen immunotherapy (SIT) treated (closed bar) and untreated (open bar) subjects with allergic rhinitis.

The present data show that 53.1% (n = 69) of subjects with allergic rhinitis who were not treated with allergen immunotherapy developed asthma at the end of follow up, while only 41.6% (n = 84) of subjects with allergic rhinitis who took allergen immunotherapy were diagnosed with asthma (Figure 3). Our study shows that there was a significant 12% reduction in the prevalence of physician-diagnosed asthma in adults with allergic rhinitis with a number needed to treat (NNT) of 8.7. This relationship between immunotherapy and the incidence of asthma was still observed when the duration of rhinitis was included in the model (data not shown).

Out of the 232 allergic rhinitis subjects with a positive skin prick test for *Parietaria*, specific immunotherapy for at least 3 consecutive years was administered to 149 subjects (64.2%). Of the allergic rhinitis subjects with a positive skin prick test for HDM, 64 out of 107 (59.8%) received specific immunotherapy for at least 3 consecutive years. Out of the 53 subjects with a co sensitivity towards *Parietaria *and HDM, 38 received *Parietaria *immunotherapy and 15 were treated with HDM immunotherapy. Thus patients on immunotherapy were either treated with *Parietaria *allergen or mites allergen alone.

**Figure 4:**
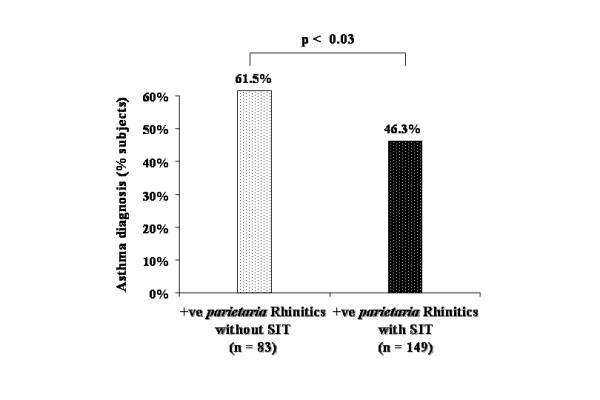
Percentage of new onset asthma by the end of the study in *Parietaria *immunotherapy (SIT) treated (clear pointed bar) and untreated (dark pointed bar) allergic rhinitic subjects with positive skin prick test to *Parietaria*.

**Figure 5:**
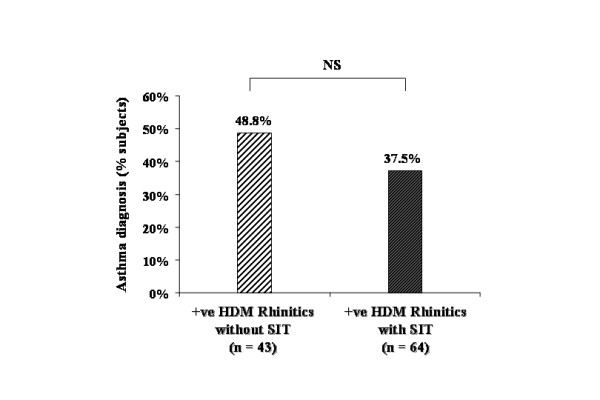
Percentage of new onset asthma by the end of the study in HDM immunotherapy (SIT) treated (clear hatched bar) and untreated (dark hatched bar) allergic rhinitic subjects with positive skin prick test to HDM.

The present data show that 61.5% (n = 51 *out of 83*) of positive *Parietaria *subjects with allergic rhinitis but who were not treated with *Parietaria *immunotherapy developed asthma at the end of follow up while only 46.3% (n = 69 *out of 149*) of subjects with allergic rhinitis who took *Parietaria *immunotherapy were diagnosed with asthma (Figure 4) (Chisq = 4.89, p = 0.027). The NNT for this subgroup was 6.6. In contrast, 48.8% (n = 21 *out of 43*) of positive HDM subjects with allergic rhinitis who were not treated with HDM immunotherapy developed asthma at the end of follow up, while only 37.5% (n = 24 *out of 64*) of subjects with allergic rhinitis who received specific immunotherapy were diagnosed with asthma (Figure 5) (Chisq = 1.35, p = 0.24). The NNT for this subgroup was 8.8.

In the univariate analyses, treatment with allergen immunotherapy during follow up was inversely related to development of asthma after 10 years (OR, 0.63; 95%CI, 0.40–0.98) (Table 2). This association became stronger after adjusting for all other variables in the multivariate model (Table 2). When carrying out univariate analyses for the relationship between immunotherapy and asthma among those with *Parietaria *positive skin prick testing the OR was 0.54 (95% CI 0.31–0.94), whereas for those with HDM positive skin prick testing the OR was 0.63 (0.29–1.38), but was not significant statistically.

## Discussion

In this retrospective cohort study we found that allergic rhinitis is strongly predictive of the development of asthma even after adjustment for other risk factors for asthma. Other significant risk factors for the development of asthma were female sex, pet ownership, and family history of allergic diseases. With the exception of *Parietaria judaica*, sensitization to the allergens tested did not predict long-term asthma diagnosis. In addition, we have shown for the first time that treatment with allergen immunotherapy decreases the incidence of asthma in adults with allergic rhinitis.

The effect of rhinitis on the onset of asthma has been already investigated in longitudinal studies. Huovinen et al. [17] found that hay fever increased the risk of development of asthma during a 15-year follow-up period by 4 times among adult men and by 6 times among women. However, no information on atopic status was available. Similarly, in the cohort of Brown University freshmen [14] both allergic rhinitis and positive skin test responses increased the risk of development of asthma by about 3 times. This is in support of our findings, although the risk from our data was much higher. Data from large population-based studies clearly show that rhinitis is a risk factor for asthma among subjects with negative, as well as positive, skin test responses thus suggesting that rhinitis and asthma are not associated simply because they share atopy as a common risk factor [15,16,18,30].

In this study, some of the factors that have been normally considered important risk factors for the development of asthma were examined. As one would predict, the female sex, the presence of pets in the home and a positive family history of allergic diseases were all predictive of new onset asthma in our study population of Sicilians. Our findings are in agreement with the results from most studies of adults showing that asthma is more prevalent in women than in men [31,32] and that family history of allergy is consistently identified as an important risk factor for asthma [33]. In keeping with the role of indoor allergens from domestic animals as an important risk factor for asthma and asthma-related symptoms [34,35], we have shown that the presence of pets in the home was significantly predictive of new onset asthma in individuals with allergic rhinitis.

Sensitization to allergen has been shown to be one of the strongest determinants of asthma, and individuals with a predisposition for atopy are at higher risk [36]. Of all known common allergens, the house dust mite is known to be strongly implicated as a potential cause of asthma [37,38]. However, in this population of Sicilians, house dust mite sensitization was not significantly predictive of new onset asthma. Instead, we have been able to show for the first time that (of all allergens tested) positive sensitization to *Parietaria judaica *markedly increased the risk of developing asthma. The reasons for this discrepant result are not known, but are probably related to the peculiar characteristics of the inhalant allergen type. The *Parietaria *pollen is widespread in the Mediterranean area with a very high frequency of sensitization (up to 80% in Sicily) and its long persistence in the atmosphere (*Parietaria *pollen season in Sicily ranges from February to October) is often responsible for most perennial symptoms [39]. In contrast to mite allergens, *Parietaria *pollen has very strong allergenic properties and often reaches very high peak levels during its season [39,40].

It is unclear why a large proportion of individuals with atopy and rhinitis eventually progress to bronchial asthma. Although atopy *per se *carries an increased risk for subsequent development of asthma in rhinitic individuals, it is likely that chronic exposure to airborne allergens is important. In our study population, up to 70% of rhinitic subjects were allergic to *Parietaria *pollen. In Sicily, *Parietaria*-sensitive subjects with allergic rhinitis are likely to be exposed to very high allergen levels, and this high allergenic load may promote progression to bronchial inflammation and asthma. The findings of our recent work in non-asthmatic subjects with allergic rhinitis monosensitized to *Parietaria judaica *shows a substantial increase in non-invasive surrogate markers of bronchial inflammation during periods of seasonal exposure to *Parietaria *pollen [41], thus suggesting that ongoing exposure to *Parietaria *pollen is closely associated with inflammatory changes in the bronchial airways of subjects with allergic rhinitis, that may advance to clinical asthma.

Another significant finding of the present study is that treatment with allergen immunotherapy reduces the development of asthma in adults with allergic rhinitis. This association has been investigated here for the first time among adults. In support of our findings, clinical research studies have suggested that when allergen immunotherapy is introduced to individuals with allergic rhino-conjunctivitis, the development of asthma may be halted. The pioneering study of Johnstone and Dutton [42] showed that 28% of children receiving allergen vaccination developed asthma in compared to 78% of placebo-treated children. The Preventive Allergy Treatment (PAT) study in children with grass or birch pollen rhino-conjunctivitis [21] has been instrumental in providing encouraging evidence to support the notion that specific allergen immunotherapy may stop the development of asthma. From six paediatric allergy centres in Austria, Denmark, Finland, Germany and Sweden, 205 children with moderate to severe hay fever symptoms were randomly assigned either to receive immunotherapy for 3 years, or to an open control group. By the end of the study, the actively treated children developed significantly fewer asthma symptoms. However, in children, wheezing and coughing from non-asthmatic respiratory illness can mimic asthma. The results from the 6 centres were not consistent and there were a small number of children in each centre. Furthermore, 20% of the study population were diagnosed with asthma at baseline and apparently were not excluded. In a recent randomized, placebo-controlled 3-year study of allergen immunotherapy in non-asthmatic, rhinitic adults monosensitized to *Parietaria *pollen, we reported that 47% of patients in the placebo group developed asthma symptoms by the end of the study, as opposed to only 14% of those treated with immunotherapy [23]. However, the small sample size (n = 29), including only those sensitized to *Parietaria*, limited our power to detect a statistically significant change between the two groups.

The observed effect of allergen IT in reducing the onset of new asthma cases is of clinical importance. Our study shows that there was a significant 12% reduction in the prevalence of physician-diagnosed asthma in adults with allergic rhinitis, with a number needed to treat (NNT) of 8.7; which is better than that of 10 obtained in the recent Childhood Asthma Prevention Study with omega-3 fatty acid supplementation and house dust mite allergen avoidance [43]. To our knowledge there is no other asthma prevention study that can exhibit a greater effect. Moreover, *Parietaria *immunotherapy appears to reduce development of asthma even further. Considering the high prevalence of *Parietaria *sensitivities in our area and the importance of positive sensitization to *Parietaria *as a major independent risk factor for the development of asthma in this study population, we did secondary analysis for the effects of allergen specific immunotherapy among those individuals with positive sensitivity to *Parietaria *and those with positive sensitivity to HDM. This analysis clearly shows that treatment with *Parietaria *immunotherapy reduces development of asthma in adults with allergic rhinitis, with a calculated NNT of 6.6. In contrast, treating positive HDM subjects with allergic rhinitis with HDM immunotherapy failed to reduce the rising incidence of asthma in this subgroup. The observed disparity in responses to different allergen extracts for IT in the present study may provide an additional explanation for the inconsistency in the results from the 6 centres in the PAT-study [21].

Our study has the advantage of a relatively long follow up period of 10 years. The cohort approach minimizes the possibility of reverse causality that may be encountered in case-control studies, where it is not possible to know if the asthma began after or before the exposure, or in this case allergic rhinitis. Another advantage of this study is the rigorous clinical assessment of asthma diagnosis prior to exclusion at baseline and for its diagnosis as an outcome at the end of follow up. Failing to diagnose actual asthma cases at baseline would have introduced systematic bias, which could affect the results by either increasing or decreasing the observed OR. On the other hand, missing the diagnosis of asthma at the end of follow up would have attenuated the observed OR. Even with all the instruments and expertise available for this study, we had to exclude 39 cases because asthma diagnosis was unclear. The fact that the study subjects were examined by the same respiratory unit at baseline and at follow up 10 years afterwards is important for standardising asthma diagnosis criteria in such a population.

It is also important to obtain an accurate history of steroid and other asthma treatments during follow up. As intranasal use of corticosteroids [44,45] has been shown to reduce asthma symptoms in patients with allergic rhinitis, we attempted to address these variables in our cohort and there was no difference between asthmatics and non-asthmatics for these treatments. This may explain the higher OR in our study compared to earlier studies.

A possible weakness of our study includes relying on medical records for the selection of the study subjects at baseline. However, all these subjects were examined and carefully diagnosed and documented in the clinic by our specialists. The lower response rate due to the change in the telephone numbers during follow up might have affected our results. However, we do not expect that this lower response is related to the diagnosis of asthma and therefore any random error introduced by lower participation is likely to attenuate rather than exaggerate the observed OR.

In conclusion, our study shows that immunotherapy can be used to reduce progression to asthma later on, especially when there are symptoms of allergic rhinitis and atopy. Allergic rhinitis seems to pose a much higher long-term risk than previously thought. The main allergy-related risk for asthma in Siciliy, and possibly other Mediterranean countries, seems to be *Parietaria *pollen rather than house dust mites. These are important findings for clinicians to help guide them in the prevention and treatment of their patients. Well conducted clinical trials may shed more light on the significance of our findings in relation to the long-term prevention of asthma.
